# Role of miR-92a-3p, oxidative stress, and p38MAPK/NF-κB pathway in rats with central venous catheter related thrombosis

**DOI:** 10.1186/s12872-020-01436-x

**Published:** 2020-03-30

**Authors:** Xiao Gan, Huihan Zhao, Yan Wei, Qingjuan Jiang, Cui Wen, Yanping Ying

**Affiliations:** Department of Cardiothoracic surgery, The First Affiliated Hospital of Guangxi Medical University, No. 6 Shuangyong Road, Nanning, 530021 China

**Keywords:** Central venous catheter, Catheter-related thrombosis, miR-92a-3p, Oxidative stress, p38 MAPK/NF-κB pathway

## Abstract

**Background:**

miR-92a-3p and oxidative stress are reportedly associated with venous thrombosis. However, the role of miR-92a-3p and oxidative stress in catheter-related thrombosis (CRT) remains ambiguous. Herein, we studied the roles of miR-92a-3p, oxidative stress, and p38-mitogen-activated protein kinase/nuclear factor kappa-B (MAPK/NF-κB) pathway in CRT.

**Methods:**

Forty-five male rats were randomly and equally divided into control, sham operation, and CRT groups. The rats were sacrificed after 10 days. Reactive oxygen species (ROS), superoxide dismutase (SOD), and malondialdehyde (MDA) levels in the serum were determined by enzyme-linked immunosorbent assay (ELISA). The expression levels of miR-92a-3p, heme oxygenase-1 (HO-1), NF-κB p65, and p38 MAPK in the venous tissues were detected with quantitative polymerase chain reaction (qPCR) and Western blot.

**Results:**

Thrombosis was observed only in the CRT group. Compared with the levels in the control and sham operation groups, ROS and MDA significantly increased in the CRT group, but SOD significantly decreased. qPCR and Western blot results showed that miR-92a-3p, HO-1, p38 MAPK, and NF-κB p65 expression was significantly upregulated in the venous tissues of the CRT group. Moreover, miR-92a-3p was positively correlated with HO-1, which was positively correlated with p38 MAPK and NF-κB p65.

**Conclusion:**

miR-92a-3p was correlated with oxidative stress in CRT. miR-92a-3p and oxidative stress contributed to endothelial dysfunction and simultaneously was associated with CRT.

## Background

Central venous catheter (CVC) facilitate the delivery of medications and blood products and provide venous access for hemodialysis, apheresis and laboratory blood draws [1, 2]. Using CVC is one of the strategies to improve the care for patients with acute or chronic diseases. However, CVC has been associated with high incidence of complications, such as thrombosis, infection, and stenosis development. Catheter-related thrombosis (CRT) is one of the most serious complications of CVC, and this complication can lead to pulmonary embolism, recurrent deep venous thrombosis, post-thrombotic syndrome, and sepsis [3, 4]. A large and comprehensive system review has shown that the incidence rate of CRT is 0–64.5% [5]. The majority of CRT patients is asymptomatic, and only 1–5% of patients manifest symptoms [6]. Hence, CRT is an urgent concern because it may promote chronic venous occlusion resulting in loss of vascular access. This condition may increases morbidity, mortality, length of hospital stay, and cost of healthcare [1, 7, 8].

MicroRNAs (miRNAs) are endogenous, single-stranded, and noncoding RNAs that play key roles in cell differentiation and proliferation. Researches of miRNAs in angiogenesis and thrombosis have been paid widespread attention [9]. The miR-17–92 cluster (miR-17, miR-18a, miR-19a/b, miR-20a, and miR-92a) is highly expressed in endothelial cells, which regulate vascular endothelial function [10–12]. miR-92a can promote atherosclerosis by upregulating proinflammatory signaling in endothelial cells [13]. The level of the miRNA-17-92 cluster increases 24 h after coronary occlusion [14]. In addition, miR-92a-3p is upregulated in the vascular tissues in a rat model of deep vein thrombosis [15]. miR-92a-3p is very sensitive to changes in shear stress. Blood flow velocity and shear stress are considerably decreased after the placement of a catheter.

The placement of a catheter in the vein can reduce local blood flow velocity by approximately 60% [16], which upregulates miR-92a expression and induces oxidative stress in endothelial cells [13]. Oxidative stress results from the imbalance between reactive oxygen species (ROS) and antioxidant defense systems and the subsequent imbalance between prooxidants and antioxidants [17]. Oxidative stress regulates physiologically the endothelial dysfunction, vascular remodeling, and inflammation [18]. Inhibition of miR-92a can hinder oxidative stress, inflammation, and apoptosis but promote angiogenesis [19]. This inhibition effect attenuates oxidative stress and improves endothelial function by enhancing HO-1 expression [20]. In addition, p38 MAPK/NF-κB is an ROS-sensitive signaling pathway, and an increase in ROS can promote endothelial cell apoptosis and activate inflammation, leading to thrombosis [21, 22].

However, whether miR-92a-3p, oxidative stress, and oxidative stress-mediated pathway of p38 MAPK/NF-κB regulates vascular system dysfunction in CRT remains unknown. What’s more, their relationship in CRT remain unclear. We hypothesized that miR-92a-3p may have correlation with oxidative stress and they contribute simultaneously to CRT. Therefore, the current study was designed to investigate whether miR-92a-3p and oxidative stress is associated with CRT.

## Methods

### Animal

Forty-five male Sprague-Dawley rats (age: 8 weeks, weight: 200 ~ 250 g) were provided by Experimental Animal Center, Guangxi Medical University, and the experimental animal breeding license number was SCXK Gui 2014–0002. The rats were allowed free access to laboratory rat chow and water in a 12 h/12 h light/dark cycle. This study was performed according to Laboratory Animal Guideline for the Ethical Review of Animal Welfare (China) and approved by Animal Care and Welfare Committee at Guangxi Medical University. The protocols conformed to the Guide for the Care and Use of Laboratory Animals by the US National Institutes of Health (NIH Publication No. 85–23).

### CVC catheterization

**Fig. 1:**
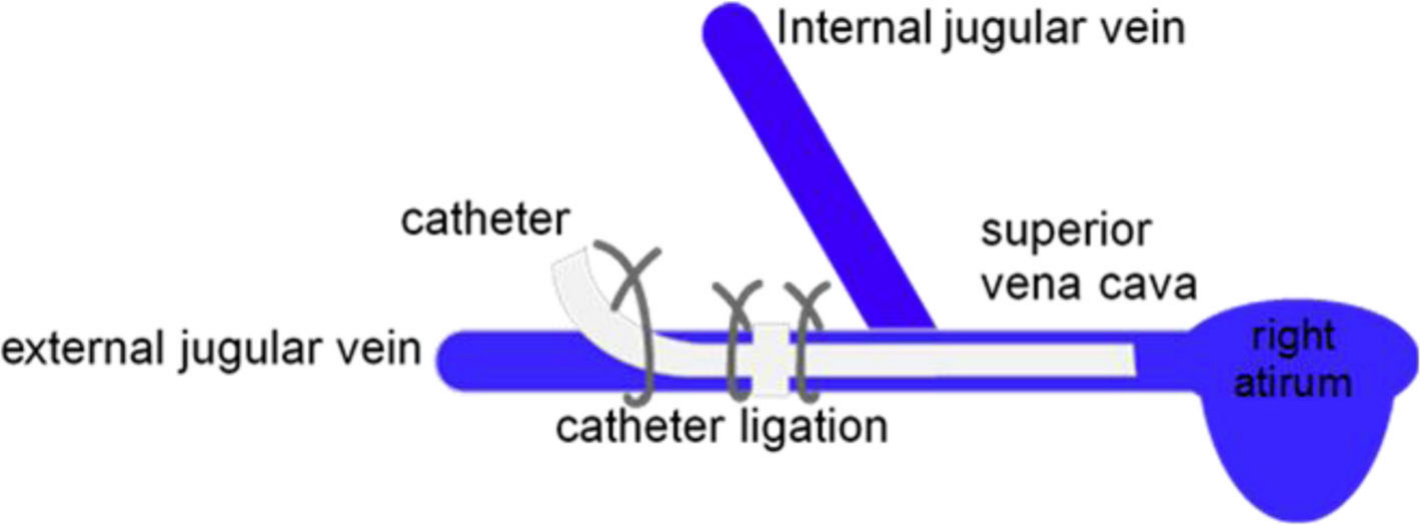
Placement of a catheter into the external jugular vein of the rats. Both nodes of the catheter were sutured to secure the catheter in place. The extravascular catheter was fixed to the subcutaneous tissue with a suture

A total of 45 rats were randomly divided into the control, sham, and CRT groups, with 15 rats per group. The catheter (Skillsmodel, Beijing, China) was sterilized and flushed with heparin solution (100 IU/mL) before surgery. The rats were weighed using a digital scale. The CRT group was anesthetized by intraperitoneal injection of 3% pentobarbital sodium. Afterwards, the skin of the right neck was shaved and disinfected with povidone iodine. All surgical instruments were sterilized using steam sterilization, and disposable sterile fenestrated sheet was used to create a sterile field around the neck. A ventral cervical skin incision (1.5 cm) was made from the lower mandible to slightly anterior to the level of the clavicle. The right external jugular vein (EJV; 1 cm) was isolated by blunt dissection from the surrounding connective tissue. After the application of one small drop of 0.5% lidocaine, the distal region of the EJV was ligated with a 3–0 silk suture for proximal region occlusion. A small venotomy was made through which the catheter was introduced into the jugular vein through the EJV and then advanced by 3.5 cm into the superior vena cava. Then, the catheter was secured to the vein by silk sutures to prevent movement (Fig. 1). Afterwards, 2 mL of the heparin solution (100 U/mL) was used to lock the catheter, and the plug was inserted. The incision sites were closed by sutures and treated with mopiroxacin ointment. The animals were moved to the animal facility after recovery from anesthesia. Anticoagulant therapy was not used in this rat model for 10 days. No catheter was inserted in the sham operation group. The surgical method was the same as that of the CRT group. The necks of the rats were sutured after the exposure of the EJV. The control group was not treated.

### Histology

After a catheterization period of 10 days, 45 rats were followed by euthanized with pentobarbital overdose. In CRT group, catheterized veins were obtained and fixed in 10% neutral buffered formalin overnight. After fixation by immersion, the catheters were removed slowly from the veins before dehydration. The veins obtained from the control and sham operation groups were ipsilateral to the CRT group. Then, the tissues were embedded in paraffin, and 5 μm cross-sections were cut. All tissue sections were stained with hematoxylin and eosin (H&E) and evaluated by a pathologist for thrombosis. The images of the stained sections were digitized using a microscope (BX53, Olympus, Japan). Digital managing software (cellSens standard) was used to capture images.

### Elisa

Blood samples from the abdominal aortas of the euthanized rats were collected into dry vacuum tubes without additives. The sera were collected by centrifugation at 4000×*g* for 5 min. ELISA kits were used by following the manufacturer’s protocol to detect the ROS (ml0262881, Mlbio, Shanghai, China), MDA (ml003180, Mlbio, Shanghai, China), and SOD (ml059387, Mlbio, Shanghai, China) levels. The standard concentrations of ROS were 480 U/mL, 240 U/mL, 120 U/mL, 60 U/mL, 30 U/mL, 15 U/mL. The standard concentrations of MDA were 4.8 nmol/mL, 2.4 nmol/mL, 1.2 nmol/mL, 0.6 nmol/mL, 0.3 nmol/mL, 0.15 nmol/mL. The standard concentrations of SOD were 10 ng/mL, 5 ng/mL, 2.5 ng/mL, 0.625 ng/mL, 0.3125 ng/mL.

### Quantitative polymerase chain reaction

Before the qPCR, the catheter in the CRT group was removed from the vein. To examine the relative mRNA expression of miR-92a-3p, HO-1, p38 MAPK, and NF-κB p65, the total RNA was extracted using miRcuet miRNA isolation kit (TIANGEN, Beijing, China) and TRIzol reagent (Solarbio, Beijing, China). Reverse transcription was accomplished with miRcuet Plus miRNA First-strand cDNA kit (TIANGEN, Beijing, China) and Monscript™ RT III all-in-one mix (Monad, Wuhan, China). The reverse transcription products were amplified with miRcuet Plus miRNA qPCR kit (SYBR GREEN) (TIANGEN, Beijing, China) and MonAmp™ SYBR® Green mix (Monad, Wuhan, China) according to the manufacturer’s instructions. The relative expression levels of the target genes were determined using 2^-△△CT^. U6 and GAPDH were used as the internal controls to identify the miRNA and mRNA, respectively.

The sequences of primers were listed as follows:

miR-92a-3p-F: 5′-ATAACGTGAACAGGGCCGG-3′,

miR-92a-3p-R: 5′-CAGTGCGTGTCGTGGAGT-3′,

HO-1-F: 5′-TCTGCAGGGGAGAATCTTGC-3′,

HO-1-R: 5′-TTGGTGAGGGAAATGTGCCA-3′,

p38 MAPK-F: 5′-GATAATGCGTCTGACGGGGA-3′,

p38 MAPK-R: 5′-ATCCGAGTCCAAAACCAGCA-3′,

NF-ΚB p65-F: 5′-CATGGATCCCTGCACACCTT-3′,

NF-κB p65-R: 5′-CTCAGCATGGAGAGTTGGCA-3′,

GAPDH-F: 5′-AGTGCCAGCCTCGTCTCATA-3′,

GAPDH-R: 5′-GATGGTGATGGGTTTCCCGT-3′,

U6-F: 5′-CTCGCTTCGGCAGCACA-3′,

U6-R: 5′-AACGCTTCACGAATTTGCGT-3′.

### Western blot

The catheter in the CRT group was removed from the vein. The venous tissues of rats in each group were lysed with RIPA lysate and centrifuged at 12000 rpm for 15 min at 4 °C. The supernatants were then obtained. After measuring the protein concentrations, the protein sample was subjected to SDS-PAGE and then transferred to a polyvinylidene difluoride (PVDF) membrane. The membrane was then sealed in 8% skim milk for 3 h and then incubated overnight at 4 °C with antibodies of HO-1 (1:1000, ab13248, Abcam, USA), NF-κB p65 (1:1000, ab16502, Abcam, USA), NF-κB p-p65 (1:1000, ab86299, Abcam, USA), p38 MAPK (1:1000, ab170099, Abcam, USA), p-p38 MAPK(1:1000, ab47363, Abcam, USA), and β-actin (1:1000, ab8227, Abcam, USA). The blots were washed thrice with TBST (T1081, Solarbio, Beijing, China) and then incubated with a 1:3000 dilution of secondary goat anti-mouse IgG H&L (ab205719, Abcam, USA) for 1 h. The protein membranes were stained using an ECL kit (Solarbio, Beijing, China), and fluorescence was determined using an imaging system (Tanon, Shanghai, China). Levels of proteins were normalized to that of β-actin.

### Statistical analysis

All data were expressed as the means ± standard deviation of at least three repeated experiments, and statistical analyses were conducted using SPSS 22.0 software (IBM SPSS, USA). Pearson correlation analysis was used to examine the correlation. One-way ANOVA with a Tukey post-hoc test was performed for multiple comparisons, and *p* < 0.05 was considered statistically significant.

## Results

### CRT in the EJV of rats

**Fig. 2:**
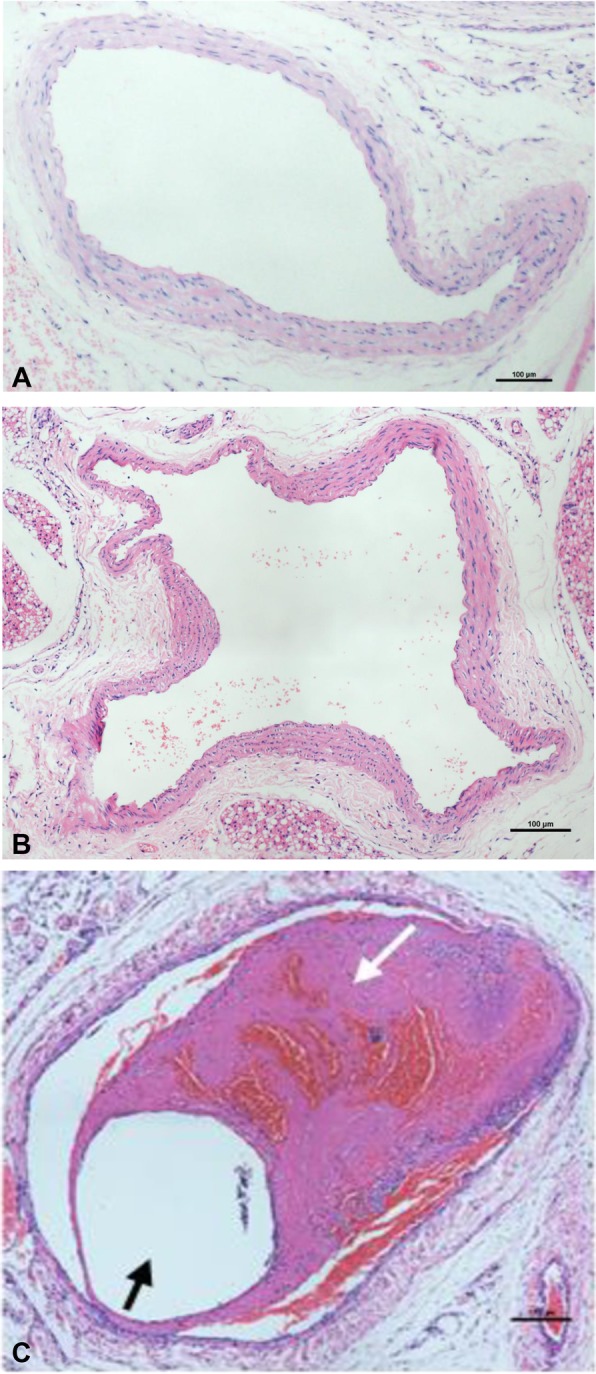
Histology of EJV in the (**a**) control, (**b**) sham operation, and (**c**) CRT groups. Black arrows indicate the lumen of the catheter, and white arrows are indicative of CRT

The success rate of the placement of a catheter was 100% (15/15), and no prolapse was observed while the catheter was installed. All animals survived to the end of schedule. No thrombosis was observed in the control and sham operation groups. Thrombosis in the catheterized EJV was observed in the CRT group, and the successful rate of the CRT model was 100% (15/15). The difference between the CRT group compared with the control and sham operation groups was statistically significant (*p* < 0.001). The histology of EJV is shown in Fig. 2. In the control and sham operation groups, optical microscopy results show that the endothelial cells of the vein were smooth and intact, and no thrombosis was observed in the vessel lumen. By contrast, CRT was observed in the CRT group and located at the edge of the catheter. Trabecular platelets, red cells and white cells were present in the thrombus.

### Levels of oxidative stress markers

**Fig. 3:**
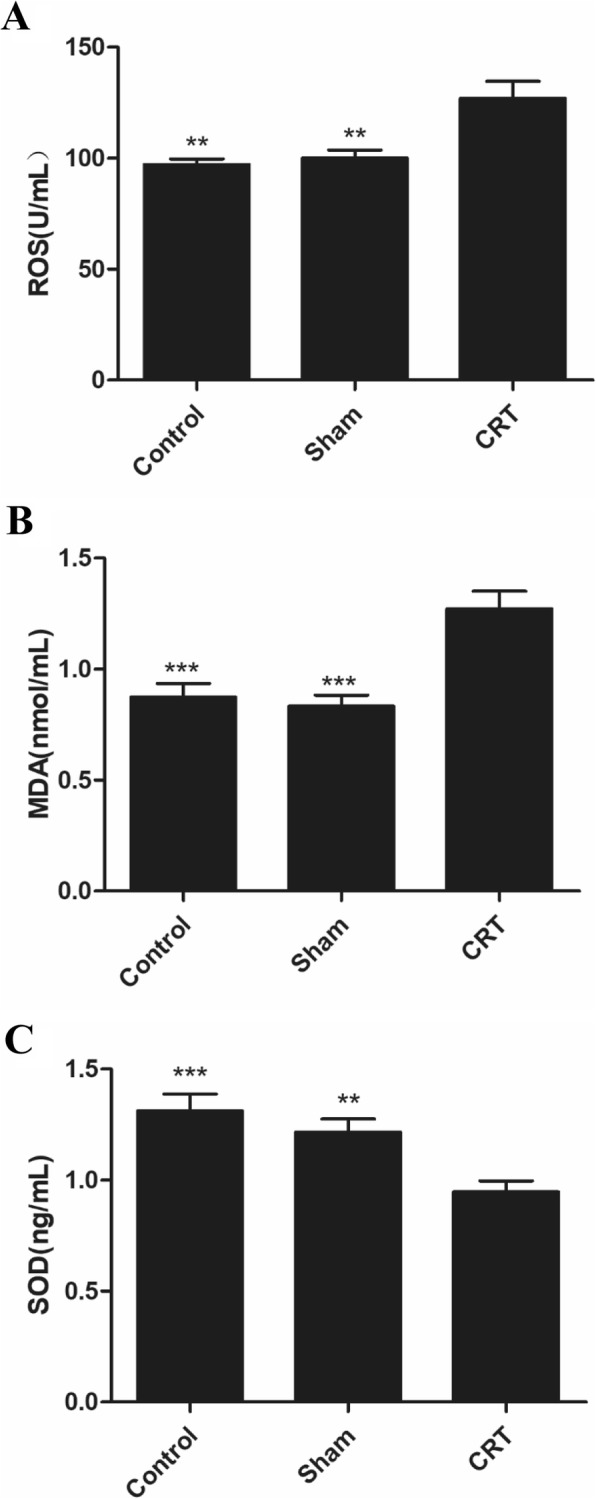
Levels of oxidative-stress markers in the sera of rats. **a** ROS; **b** MDA; and **c** SOD. ** *p* < 0.01 versus the CRT group, and *** *p* < 0.001 versus the CRT group

The levels of ROS, MDA, and SOD in the rat sera are presented in Fig. 3. The results showed that the ROS and MDA levels in the CRT group significantly increased compared with those of the control and sham operation groups, but SOD significantly decreased. The differences between the serum ROS, MDA, and SOD levels of the control and shame operation groups were nonsignificant.

### Upregulated mRNA expression of miR-92a-3p, HO-1, and p38 MAPK/NF-κB

**Fig. 4:**
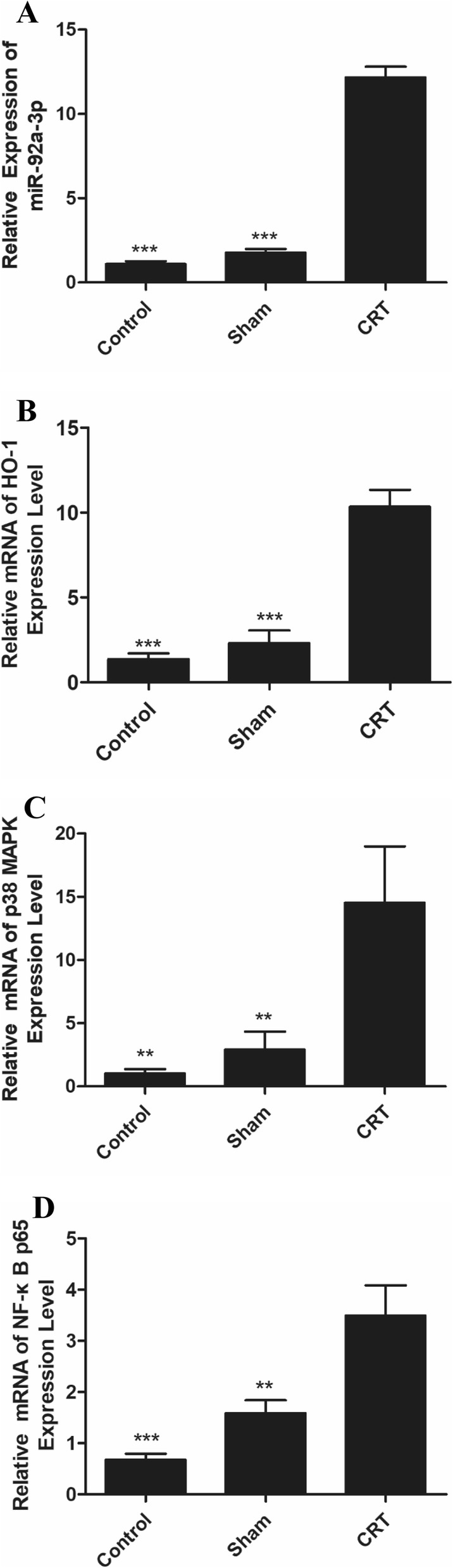
Expression of miR-92a-3p, HO-1, p38 MAPK, and NF-κB p65 mRNA was upregulated in the veins of rats with CRT. **a** miR-92a-3p; **b** HO-1; **c** p38 MAPK and **d** NF-κB p65. ** *p* < 0.01 versus CRT group, and *** *p* < 0.001 versus CRT group

The miR-92a-3p, HO-1, p38 MAPK, and NF-κB p65 mRNA expression levels in the venous tissues of rats were detected with qPCR. Figure 4 shows significant upregulation of miR-92a-3p, HO-1, p38 MAPK, and NF-κB p65 mRNA expression levels in the veins of the CRT group.

### Protein expression of HO-1 and p38 MAPK/NF-κB pathway

**Fig. 5:**
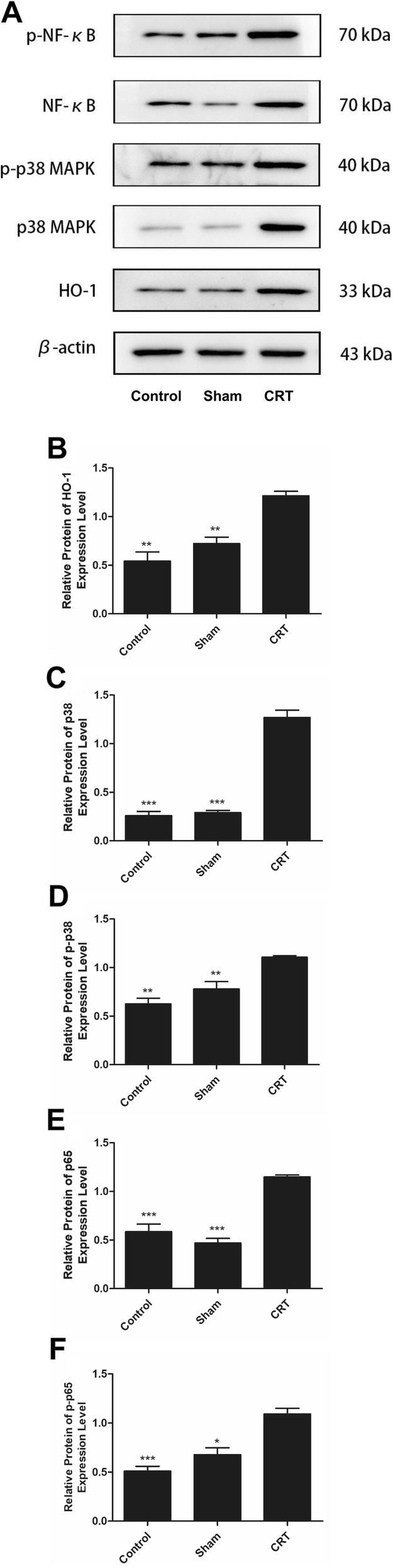
Protein expression of HO-1 and p38 MAPK/NF-κB pathway were upregulated in CRT. **a** Western blot image of HO-1, p38 MAPK, p-p38 MAPK, NF-κB p65 and p-NF-κB p65. **b**-**f** analysis of the protein expression of HO-1, p38 MAPK, p-p38 MAPK, NF-κB p65 and p-NF-κB p65. * *p* < 0.05 versus CRT group, ** *p* < 0.01 versus CRT group, and *** *p* < 0.001 versus CRT group

The protein levels of HO-1, p38 MAPK, p-p38 MAPK, NF-κB p65, and p-NF-κB p65 in the CRT group were upregulated compared with those of the control and sham operation groups (Fig. 5). High p38 MAPK expression was found in the CRT group, whereas low levels of this molecule was observed in the control and sham operation groups (Fig. 5a).

### Positive correlation between miR-92a-3p, HO-1, and p38 MAPK/NF-κB pathway in the venous tissues

Pearson correlation analysis showed that miR-92a-3p was positively correlated with HO-1 (r = 0.770, *p* = 0.015). HO-1 was positively correlated with p38 MAPK (r = 0.788, *p* = 0.035), and NF-κB p65 (r = 0.797, *p* = 0.018).

## Discussion

Thrombosis is a disease involving multiple factors and systems and a major contributor to global health burden [23]. Many approaches, such as changes in the diameter of catheter [24], tuning of catheter-to-vein ratio [25], novel catheter surface coatings [26], use of catheter-locking solutions [27] or anticoagulants [28], and handgrip exercise [29], have been investigated to prevent CRT.

Numerous reports on CRT were clinical studies. However, our study provides a new view on CRT by using an animal experiment. In the study, we inserted a catheter into the right EJV with the catheter tip in the superior vena cava by surgical operation and successfully observed CRT in the EJV after 10 days. The result was the same as that in a previous study [30]. Venous blood stasis and endothelial injury are the major reasons for CRT [31]. Endothelial cells are injured after the placement of a catheter, and collagen exposure under the endothelium causes platelet adhesion and aggregation and activates factor XII. Concomitantly, the injured endothelium releases tissue factor, activates factor VII, and initiates intrinsic and extrinsic coagulation processes [32]. The channel for the blood flow becomes constricted, and blood velocity decreases after catheterization [33]. When blood velocity is decreased, the platelets enter the side stream, which greatly increases the possibility of adhering the intima of blood vessels. This process contributes to thrombosis.

Furthermore, the role of miR-92a-3p and oxidative stress in CRT has not been reported. In this study, miR-92a-3p, oxidative stress, and p38 MAPK/NF-κB pathway were significantly related to CRT. In addition, miR-92a-3p was positively correlated with HO-1, which was positively correlated with p38 MAPK and NF-κB p65. miR-92a-3p, which is mediated by the blood flow shear stress, regulates the expression of the endothelial cell eNOS and impair eNOS-NO bioavailability, resulting in vascular injury and contributing to atherosclerosis [13, 34, 35]. The reduced velocity or occlusion of blood in the veins upregulated miR-92a expression and induced oxidative stress in endothelial cells, leading to endothelial cell inflammation and dysfunction [13]. Oxidative stress can activate MAPK/NF-κB, mediate endothelial cell apoptosis, promote the expression of tissue factor and thrombocyte secretion, and regulate venous thrombosis [21]. Overexpression of miR-92a causes endothelial dysfunction and suppresses HO-1 expression in the endothelial cells [20]. In addition, MAPKs and NF-κB pathways are the major regulators of HO-1 expression after exposure to extracellular stimuli [36–38]. However, the forward and reverse targets for the development of HO-1 to regulate thrombosis are not fully explored. Therefore, HO-1 may be the target of miR-92a-3p and p38 MAPK/NF-κB pathway. miR-92a-3p may regulate HO-1/p38 MAPK/NF-κB pathway and result in CRT. However, further studies should be performed to identify the underlying mechanisms. In addition, miR-92a-3p was correlated with oxidative stress in CRT. These molecules play a key role during CRT. A model for predicting high-risk CRT patients could be established by using changes in the oxidative-stress markers in the plasma. According to the results from the model, CVC patients can take measures to prevent CRT as early as possible.

This study provides a new perception on animal experiment in CRT. The process is quite novel and promising in future translational research, especially for patients in the ICU and cancer patients undergoing chemotherapy. However, this study has some limitations. One limitation was that the thrombosis was only evaluated through pathology. Another limitation was the exclusion of the activation of platelets, and the coagulation system may contribute to CRT. Further studies should be performed by our team to reveal the mechanism among miR-92a-3p, oxidative stress, and oxidative stress-mediated pathways in CRT.

## Conclusion

miR-92a-3p, oxidative stress, and p38 MAPK/NF-κB were found to contribute to CRT. Positive correlation was observed among these molecules. miR-92a-3p and oxidative stress may contribute simultaneously to CRT.

## Data Availability

The datasets used and/or analysed during the current study are available from the corresponding author upon reasonable request.

## Abbreviations

CVC: Central venous catheter
CRT: Catheter-related thrombosis
ROS: Reactive oxygen species
SOD: Superoxide dismutase
MDA: Malondialdehyde
HO-1: Heme oxygenase-1
miR-92a-3p: MicroRNA-92a-3p
P38 MAPK: p38-mitogen-activated protein kinase
NF-κB: Nuclear factor kappa-B
ELISA: Enzyme-linked immunosorbent assay
qPCR: Quantitative polymerase chain reaction
